# Changes in Key Mitochondrial Lipids Accompany Mitochondrial Dysfunction and Oxidative Stress in NAFLD

**DOI:** 10.1155/2021/9986299

**Published:** 2021-06-27

**Authors:** Manon Durand, Marine Coué, Mikaël Croyal, Thomas Moyon, Angela Tesse, Florian Atger, Khadija Ouguerram, David Jacobi

**Affiliations:** ^1^Université de Nantes, CHU Nantes, CNRS, INSERM, L'institut du Thorax, F-44000 Nantes, France; ^2^Université de Nantes, CHU Nantes, INRAE, UMR1280, Physiopathologie des Adaptations Nutritionnelles (PhAN), Institut des Maladies de l'Appareil Digestif (IMAD), Centre de Recherche en Nutrition Humaine Ouest (CRNH-O), F-44093 Nantes, France; ^3^Université de Nantes, CHU Nantes, Inserm, CNRS, SFR Santé, Inserm UMS 016, CNRS UMS 3556, F-44000 Nantes, France; ^4^Centre de Recherche en Nutrition Humaine Ouest (CRNH-O) Mass Spectrometry Core Facility, F-44000 Nantes, France

## Abstract

Nonalcoholic fatty liver disease (NAFLD) is a dysmetabolic hepatic damage of increasing severity: simple fat accumulation (steatosis), nonalcoholic steatohepatitis (NASH), and hepatic fibrosis. Oxidative stress is considered an important factor in producing hepatocyte injury associated with NAFLD progression. Studies also suggest a link between the accumulation of specific hepatic lipid species, mitochondrial dysfunction, and the progression of NAFLD. However, it is unclear whether mitochondrial lipid modifications are involved in NAFLD progression. To gain insight into the relationship between mitochondrial lipids and disease progression through different stages of NAFLD, we performed lipidomic analyses on mouse livers at different stages of western diet-induced NAFLD, with or without hepatic fibrosis. After organelle separation, we studied separately the mitochondrial and the “nonmitochondrial” hepatic lipidomes. We identified 719 lipid species from 16 lipid families. Remarkably, the western diet triggered time-dependent changes in the mitochondrial lipidome, whereas the “nonmitochondrial” lipidome showed little difference with levels of hepatic steatosis or the presence of fibrosis. In mitochondria, the changes in the lipidome preceded hepatic fibrosis. In particular, two critical phospholipids, phosphatidic acid (PA) and cardiolipin (CL), displayed opposite responses in mitochondria. Decrease in CL and increase in PA were concurrent with an increase of coenzyme Q. Electron paramagnetic resonance spectroscopy superoxide spin trapping and Cu^2+^ measurement showed the progressive increase in oxidative stress in the liver. Overall, these results suggest mitochondrial lipid modifications could act as an early event in mitochondrial dysfunction and NAFLD progression.

## 1. Introduction

Nonalcoholic fatty liver disease (NAFLD) is reaching epidemic proportions, affecting a quarter of the world's adult population [1]. NAFLD begins with an accumulation of cellular fat (steatosis), progresses to hepatocellular injury with inflammation (nonalcoholic steatohepatitis (NASH)), and culminates in hepatic fibrosis, a cause of liver cirrhosis and hepatocellular carcinoma [2]. The current mechanistic view of the progression of simple steatosis into NASH proposes that exceeding the elimination capacity of free fatty acids in hepatocytes contributes to the formation of lipotoxic species, endoplasmic reticulum (ER) stress, and maladaptive responses of mitochondria (mitochondrial dysfunction) [3–5].

As the power house of hepatocytes, mitochondria play a major role in oxidative metabolism and normal function of the liver. In the early stages of NAFLD, namely, simple steatosis, mitochondrial respiration increases to adapt to the higher substrate availability and increased ATP demand. No defects were observed in the respiratory function of liver mitochondria isolated from *ob/ob* mice with hepatic steatosis [6]. The capacity of isolated liver mitochondria to oxidize fatty acids was even increased in *ob/ob* mice [6]. Humans with simple steatosis and insulin resistance show elevated mitochondrial hepatic fatty acid oxidation and respiratory function when measured noninvasively by metabolite labeling *in vivo* [7, 8] and even *ex vivo* after isolating mitochondria from liver biopsies [9]. As hepatocytes store more lipids and reach full storage capacity, free fatty acid-mediated toxicity damages mitochondria. In the transition to NASH, mitochondrial respiration is decreased and reactive oxygen species (ROS) are increased. The adaptation of hepatic mitochondrial function in humans to simple steatosis is lost in steatohepatitis [7].

The lipid composition of mitochondrial membranes is pivotal to maintain mitochondrial structure and function [10]. The proximity of mitochondrial phospholipids known as cardiolipins (CLs) to the electron transport chain (ETC) provides the lipophilic environment necessary for oxidative phosphorylation [11]. CLs maintain supercomplexes [12] and regulate the transport of electrons from complex I to ubiquinone, the oxidized state of coenzyme Q (CoQ) [13]. Notably, in NAFLD, changes in CL [14] and CoQ [15, 16] contribute to the altered activity of respiratory chain complexes and oxidative stress. Accumulation of hepatic CL and CoQ in NAFLD patients has been interpreted as an early adaptive mechanism to preserve mitochondrial function [17].

Despite all this recent progress, it is still unclear whether mitochondrial dysfunction is involved in NAFLD progression or whether it is a consequence of cellular stress or fibrosis. Preclinical models for NAFLD are increasingly evaluated on the basis of “omics” features, rather than on histology alone [2]. Hepatic mitochondrial lipidome is accessible [18], and its physiological changes can be assessed [19]. However, the hepatic mitochondrial lipidome alterations during NAFLD progression are unknown. We hypothesized that hepatic mitochondria undergo specific alterations during NAFLD evolution that could be causative of mitochondrial dysfunction. In this respect, we aimed to study the evolution of liver mitochondrial lipidome and oxidative stress in a diet-induced NAFLD mouse model.

## 2. Materials and Methods

### 2.1. Animals

The local ethics committee approved the care and use of experimental animals (Pays de la Loire, France, project APAFIS#6697, compliant with directive 2010/63/EU). We studied five-week-old male C57Bl/6J mice fed *ad libitum* with either a control chow diet (A04; Safe Diets, France: 8.4% of energy from fat, no cholesterol) or a western diet (WD) for 8, 16, or 25 weeks. The WD associated a high-fat diet (U8958v250, Safe Diets, France: 45% of energy from fat and 2% cholesterol) with 42 g/L fructose (61252 from Safe Diets) in drinking water. Mice were housed five per cage under 12 h : 12 h light : dark conditions. Mice were euthanatized by exsanguination under isoflurane anesthesia.

### 2.2. Liver Steatosis and Fibrosis Quantification

A slice of the liver was fixed in 4% paraformaldehyde for 24 h before paraffin embedding. Then, 5-micron thick sections were stained with hematoxylin-eosin-saffron (HES) or 0.1% picrosirius red (area of steatosis and fibrosis) solution. The entire stained specimen was analyzed by an automatic thresholding technique using an algorithm developed in HIFIH laboratory (EA 3859, Angers, France) as previously described [20].

### 2.3. Isolation of Liver Mitochondria

For mitochondria isolation, the differential centrifugation method was used, as described previously [21], with minor modifications. All steps were on ice or at 4°C. Caudate liver lobes (≈100 mg) were rinsed, chopped with scissors, and homogenized with a Dounce tissue grinder in 1.5 mL mitochondria isolation buffer (70 mM sucrose, 210 mM mannitol, 5 mM HEPES, 1 mM EGTA, and 0.2% fatty acid BSA, pH 7.2). The homogenate was spun 8 min at 800 g. The filtered supernatants (70 *μ*M cell strainer) were spun 8 min at 8.000 g. The resulting pellet was rinsed and spun 5 min at 8000 g. The final pellet represented the mitochondria-enriched fraction. The first pellet was pooled with the supernatants of the last two centrifugations to make the “nonmitochondrial fraction.”

### 2.4. Lipidome Analysis

#### 2.4.1. Preparation of Samples

The nonmitochondrial fraction was evaporated to dryness under a nitrogen stream. The mitochondrial-enriched and nonmitochondrial pellets were resuspended in PBS. The samples were diluted at 10 mg protein/mL.

#### 2.4.2. Nontargeted Lipidomic Analysis by Mass Spectrometry

The lipids were extracted with a methanol/chloroform method [18] and MTBE [22] method. For the methanol/chloroform method, 30 *μ*L of “extracted solution” was mixed consecutively with 200 *μ*L of methanol, 400 *μ*L of dichloromethane, and 120 *μ*L of water. After 10 minutes of incubation at room temperature (RT), the mix was spun 10 min at 8000 g at 10°C, and 370 *μ*L of the lower phase was sampled. For MTBE, 50 *μ*L of “extracted solution” was successively mixed with 450 *μ*L of ice-cold methyl-*tert*-butyl-ether (MTBE, Biosolve, Netherlands), 1500 *μ*L of ice-cold methanol (Biosolve, Netherlands), and 375 *μ*L of water. The mixes were centrifuged 10 min at 10,000 g at 4°C, and 800 *μ*L of the supernatant was sampled. For the two methods, the samples were dried under a nitrogen stream. Samples were finally resuspended in 150 *μ*L acetonitrile/isopropanol/water (65/30/5, *v*/*v*/*v*) for liquid chromatography–high-resolution mass spectrometry (LC-HRMS). A quality control (QC) was prepared by pooling 20 *μ*L of each. Samples and QC were loaded in the analytical system consisting of a SYNAPT G2 HRMS Q-TOF mass spectrometer, equipped with an electrospray ionization (ESI) interface operating in positive and negative mode, and a AQUITY UPLC H-Class System (Waters Corporation, Milford, MA, USA). Five microliters of each sample was randomly injected onto a reverse-phase CSH C18 (2.1 × 100 mm; 1.7 *μ*M) column (Waters Corporation) as described previously [23]. The data was acquired and normalized using MassLynx and MarkerLynx software, respectively (version 4.1, Waters Corporation).

#### 2.4.3. Apolipoprotein E (ApoE) Measurements

ApoE was measured by liquid chromatography–tandem mass spectrometry (LC–MS/MS) as described previously [24].

#### 2.4.4. Targeted Lipidomic Analysis

Data points with zero values or with a coefficient of variation for QC ≥ 30% were excluded from the analysis. Lipid markers were extracted from variables using both LipidMaps (http://www.lipidmaps.org) and an in-house database containing reference lipid standards. All lipid markers were checked for their exact mass-to-charge ratio (*m*/*z*), their elemental compositions with a mass error of ±5 ppm, their retention time (±30 s), and their fragmentation patterns obtained by tandem mass spectrometry (Supplementary Table S1).

### 2.5. Total RNA Extraction and RT-qPCR

Pieces of the liver (≈50 mg) were homogenized in 750 *μ*L of NucleoZOL (Macherey-Nagel, Germany) and spun 5 min at 12,000 g. The supernatant was mixed with 300 *μ*L of RNase-free H_2_O, incubated 15 min, and spun 15 min at 12,000 g. For phase separation, 3.75 *μ*L of 4-bromoanisole was added to 800 *μ*L of the supernatant and incubated 5 min. After spinning 10 min at 12,000 g, the supernatant was mixed with isopropanol (1 : 1, *v*/*v*), incubated 10 min, and spun 10 min at 12,000 g. The final pellet was washed twice with 750 *μ*L of 75% ethanol and spun 3 min at 10,000 g. The RNA pellet was dried and resuspended in water.

Following reverse transcription (High-Capacity cDNA Reverse Transcription Kit, Applied Biosystems, CA, USA), we performed real-time quantitative PCR (qPCR) with PowerUp SYBR Green Master Mix (Applied Biosystems, CA, USA) on a 7900 HT Fast Real-Time PCR system (Applied Biosystems, CA, USA). The primer sequences are listed in Supplementary Table S2. The mRNA expression levels are presented as the ratio of the gene of interest and a housekeeping gene (glyceraldehyde-3-phosphate dehydrogenase (GAPDH)).

### 2.6. Electron Paramagnetic Resonance (EPR)

Pieces of the same liver lobe were used for Cu^2+^, O_2_^−^, and semiquinone radical detection on a MiniScope MS 5000 spectrometer (Freiberg Instruments, Germany).

For Cu^2+^ measurements, a pale yellow-brown opalescent colloid Fe-(DETC)_2_ was obtained by separately dissolving 15 mM of Na-DETC (Sigma-Aldrich) and 8 mM of FeSO_4_-7H_2_0 (Sigma-Aldrich) in ice-cold Krebs-HEPES buffer under nitrogen gas bubbling and mixing the two solutions immediately. The tissue was incubated for 45 min at 37°C in colloid Fe-(DETC)_2_ solution as a spin trap. Then, the samples were frozen in liquid nitrogen before spectrometry (microwave power: 10 mW, amplitude modulation: 1 mT, modulation frequency: 100 kHz, sweep time: 150 s, 3 scans). The spectra were used to detect the peak corresponding to the oxidized Copper (Cu^2+^) linked to DETC [25].

We measured O_2_^−^ levels in tissues as described previously [26]. The tissue was incubated for 45 min at 37°C in a Krebs-HEPES solution containing 1-hydroxy-3-methoxycarbonyl-2,2,5,5-tetramethylpyrrolidine (CMH, 500 mM, Noxygen; Denzlingen, Germany) as spin probe, deferoxamine (25 mM, Sigma-Aldrich), and diethyldithiocarbamate (DETC, 5 mM, Sigma-Aldrich). The sample was analyzed in liquid nitrogen (microwave power: 10 mW, amplitude modulation: 0.4 mT, modulation frequency: 100 kHz, sweep time: 60 s, 3 scans).

For semiquinone radical detection, the tissue was frozen and analyzed in liquid nitrogen (microwave power: 10 mW, amplitude modulation: 0.7 mT, modulation frequency: 100 kHz, sweep time: 180 s, 3 scans) [27].

Signals were quantified from the amplitude peaks of the spectra after baseline correction (ESR Studio software, Freiberg Instruments, Germany). All values were expressed in arbitrary units (a.u.)/CL (a.u.).

### 2.7. Statistical Analysis

First, the unsupervised analyses, principal component analysis (PCA), were performed to assess the separation of the experiment. Then, the supervised partial least square regression analyses (PLS-DA) were performed to maximize the discrimination of the groups.

All multivariate analyses were computed under R version 3.6.0 (R Development Core Team, R Foundation for Statistical Computing, Vienna, Austria; http://www.R-project.org), with Factominer and pls packages for multivariate analysis.

For quantitative data depending on diet group and duration of diet, we used a two-way analysis of variance (ANOVA) test with a subsequent Bonferroni post hoc test, and when the equal variance test failed, a two-way ANOVA on rank test with the subsequent Tukey post hoc test was used. All statistical analyses were realized with GraphPad Prism 6 and SigmaStat 4.0 software. *N* represents the number of mice used for each time point and condition; ^∗^*p* < 0.05 was considered statistically significant.

## 3. Results

### 3.1. Metabolic Phenotyping of Mice on the Western Diet

**Figure 1:**
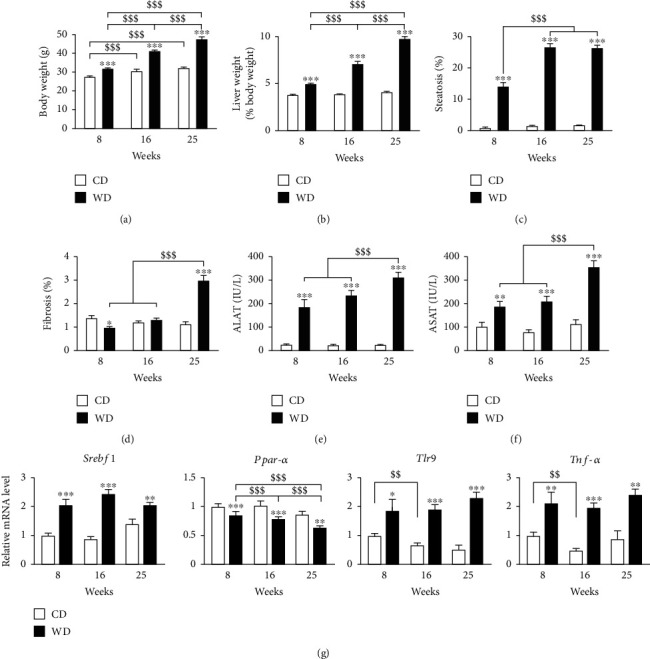
Course of metabolic phenotype under the western diet regimen. CD: control diet; WD: western diet. (a) Body weight. (b) Liver weight. (c) Hepatic steatosis. (d) Hepatic fibrosis. (e) ALAT. (f) ASAT. (g) mRNA expression of genes involved in lipid metabolism (*Srebf1*, *Pparα*) and inflammation (*Tlr9*, *Tnf-α*), from left to right. Data are expressed as mean + /−SEM; 2-way ANOVA with a subsequent Bonferroni post hoc test or 2-way ANOVA with rank with Tukey post hoc test; ^∗^*p* < 0.05, ^∗∗^*p* < 0.01, and ^∗∗∗^*p* < 0.001 versus control diet; ^$$$^*p* < 0.001 versus weeks of diet. *n* = 9–10 mice per time point and condition.

Mice with WD developed obesity, increased liver weight normalized to body weight, hepatic steatosis, ASAT, ALAT, and liver fibrosis. Notably, fibrosis was delayed, appearing at week 25 only as opposed to steatosis that was present from week 8 (Figures 1(a)–1(f)). mRNA expression of the key transcription factor for lipid synthesis, Sterol Regulatory Element-Binding Transcription Factor 1 (*Srebf1*), was increased, while that of the nuclear receptor peroxisome proliferator-activated receptor *α* (*Pparα*) was decreased. There was also an increase of mRNA levels of inflammatory pathway genes toll-like receptor 9 (*Tlr9*) and tumor necrosis factor (*Tnf-α*) with steatosis (Figure 1(g)).

CL 72 : 8 and 70 : 7, used as references for the CL family, were more extracted using MTBE method than the methanol/chloroform method (Supplementary Figure 1a). Thus, we used the MTBE method. We verified that the diet type or duration did not affect the isolation process. Based on CL retrieval, the mitochondrial fraction contained over 70% of the total mitochondria. Based on ApoE content, cytosolic contamination was below 5% (Supplementary Figure 1b).

### 3.2. NAFLD Alters the Hepatic Mitochondrial Lipidome

**Figure 2:**
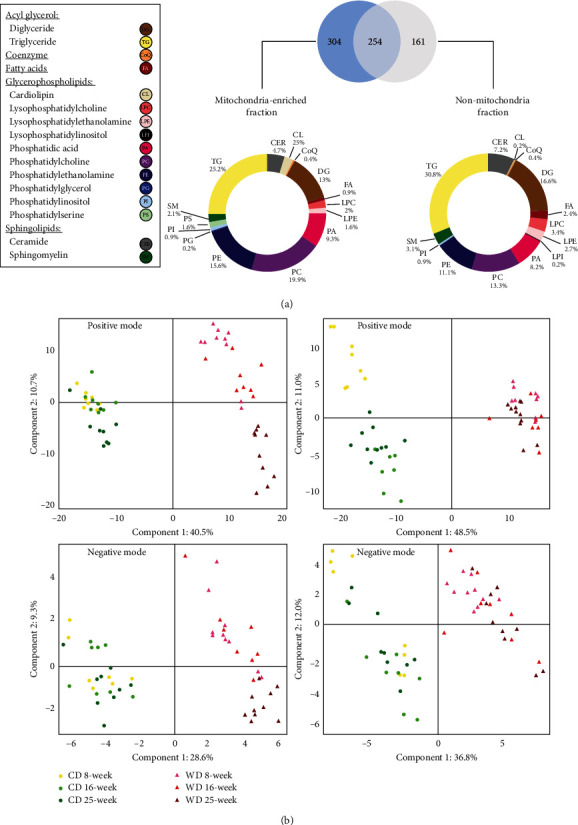
The western diet triggers time-dependent changes in the hepatic mitochondrial lipidome. (a) Venn diagram of the 719 lipid species detected in the mitochondria-enriched fraction and the nonmitochondrial fraction. The analyses were pooled for all animals included in the study. The individual species are grouped within 16 lipid families. (b) Partial least square regression analysis of lipids in the liver. Left panels: from the mitochondria-enriched fractions in positive mode (505 lipid species, top panel) and negative mode (53 lipid species, bottom panel). Right panels: from the non-mitochondria fractions in positive mode (356 lipid species, top panel) and negative mode (59 lipid species, bottom panel).

The complete raw MS results and the corresponding identified lipid species are presented in Supplementary Table 1. We identified 719 lipids from 16 different lipid families in the liver samples (Figure 2(a)). Five families (phosphatidic acid (PA), phosphatidylcholine (PC), phosphatidylethanolamine (PE), diacylglycerol (DAG), and triacylglycerol (TAG)) represented over 80% of the number of detected lipid species. For all fractions, over 90% of the lipid species were detected in the positive mode.

The PCA discriminated the CD and WD groups and showed an evolution over time for the mitochondria-enriched fractions in the WD condition (Supplementary Figure 2). The PLS-DA (a supervised method, contrary to the PCA) discriminated the CD and WD groups both for the mitochondrial and nonmitochondrial fractions (Figure 2(b)). Notably, for the mitochondrial fraction, PLS-DA showed an evolution over time only for the WD condition (Figure 2(b)), in the positive mode. This pattern was not seen in the nonmitochondrial fraction (Figure 2(b)), indicating that WD specifically affected the mitochondrial lipidome over time.

### 3.3. NAFLD Specifically Decreases Mitochondrial Cardiolipin

**Figure 3:**
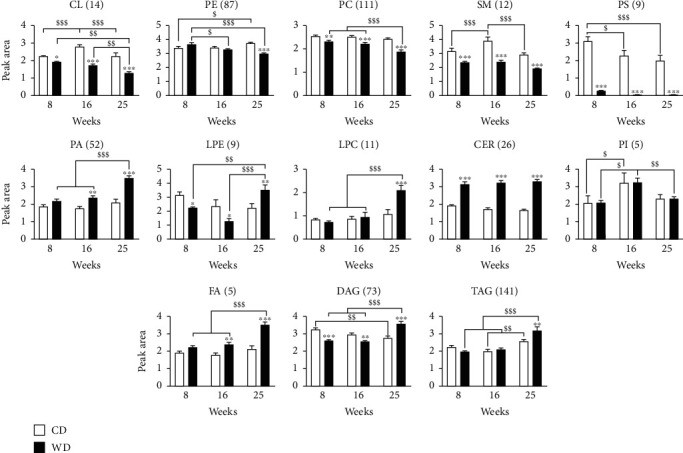
NAFLD alters mitochondrial lipid species. LC-HRMS semiquantification analysis of lipids extracted from the mitochondria-enriched fraction of the liver. After identification of the individual lipid signatures, lipids were grouped in families by adding the individual peak areas. The number of lipid species is indicated between brackets. Peak area values are arbitrary. CL: cardiolipins; PE: phosphatidylethanolamine; PC: phosphatidylcholine; SM: sphingomyelin; PS: phosphatidylserine; PA: phosphatidic acid; LPE: lysophosphoethanolamine; LPC: lysophosphatidylcholine; CER: ceramide; PI: phosphatidylinositol; FA: fatty acids; DAG: diacylglycerol; TAG: triacylglycerol. (a, b) Data are expressed as mean + /−SEM; 2-way ANOVA with subsequent Bonferroni or 2-way ANOVA with rank with Tukey *post hoc* test; ^∗^*p* < 0.05, ^∗∗^*p* < 0.01, and ^∗∗∗^*p* < 0.001 versus control diet; ^$^*p* < 0.05, ^$$^*p* < 0.01, and ^$$$^*p* < 0.001 versus weeks of diet. *n* = 7–10 mice per time point and condition.

We performed a semiquantification of the lipid families to determine the evolution of mitochondrial lipids under WD (Figure 3). CLs, PEs, and PCs significantly decreased over time, while PAs, lysophosphatidylcholines (LPC), fatty acids (FA), DAGs, and TAGs increased. SM and PS were decreased at all time points while ceramides (CER) were increased at all time points. In contrast, phosphatidylinositol (PI) was unchanged by WD over time.

### 3.4. Liver Steatosis Increases Hepatic Oxidative Stress

**Figure 4:**
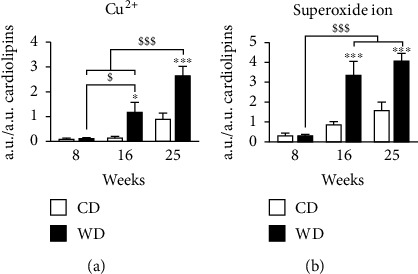
Western diet progressively increases oxidative stress in the liver. EPR was applied to a piece of the liver for the detection of (a) oxidized state of copper (Cu^2+^) and (b) superoxide ion. All values were expressed in arbitrary units (a.u.)/a.u. of cardiolipin. Data are expressed as mean + /−SEM; 2-way ANOVA with (b) subsequent Bonferroni or 2-way ANOVA on rank with (a) Tukey post hoc test; ^∗^*p* < 0.05 and ^∗∗∗^*p* < 0.001 versus control diet; ^$^*p* < 0.05 and ^$$$^*p* < 0.001 versus weeks of diet. *n* = 4–5 mice per time point and condition.

To investigate whether the alteration of mitochondrial lipids was associated with oxidative stress, we analyzed ROS levels by EPR in the whole liver. Free Cu^2+^ and O_2_^−^ levels were similar at week 8 between groups. Cu^2+^ (Figure 4(a)) and O_2_^−^ (Figure 4(b)) increased in the WD group at weeks 16 and 25 compared to the controls. For O_2_^−^, there was an increase over time in mice under CD that quadrupled between week 8 and week 25. The increase in O_2_^−^ was significantly higher in mice with WD.

### 3.5. Liver Steatosis Increases Semiquinone and Ubiquinone Levels in Mitochondria

**Figure 5:**
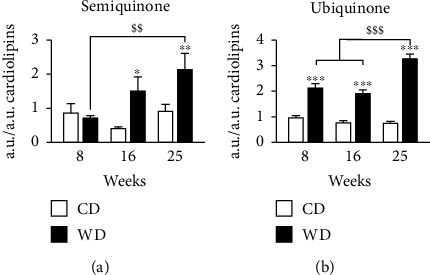
Western diet increases semiquinone and ubiquinone levels in mitochondria. (a) Semiquinone from EPR spectrometry applied to a piece of the liver. (b) LC-MS ubiquinone semiquantification analysis was performed on the lipids extracted from the mitochondria-enriched fraction of the liver. Peak area values are arbitrary. (a, b) Data are expressed as mean + /−SEM; 2-way ANOVA on rank with Tukey post hoc test; ^∗^*p* < 0.05, ^∗∗^*p* < 0.01, and ^∗∗∗^*p* < 0.001 versus control diet; ^$$^*p* < 0.01 and ^$$$^*p* < 0.001 versus weeks of diet. *n* = 7–10 mice per time point per condition.

To determine the consequences of steatosis on mitochondrial function, we performed a semiquantification of semiquinone by EPR and ubiquinone by LC-HRMS (Figure 5). Semiquinone is the intermediate molecule between the fully oxidized (ubiquinone) and the fully reduced states (ubiquinol) of coenzyme Q and participates to modulate ROS production during the electron traffic from complexes I and II to complex III of the ETC [28]. EPR analysis showed a significant increase after 25 weeks of WD for semiquinone (Figure 5(a)). In contrast to its intermediate form, ubiquinone sharply increased already from week 8 of WD (Figure 5(b)).

## 4. Discussion

We used a murine dietary model of NAFLD to demonstrate for the first time the presence of a specific evolution of the hepatic mitochondrial lipidome during NAFLD progression. The most notable modifications are a progressive decrease in CL, PE, and PC and a progressive increase in PA and CoQ (ubiquinone and semiquinone). These alterations accompany steatosis and oxidative stress but precede hepatic fibrosis.

WD induces steatosis in the short time of eight weeks [29]. The C57BL/6 mouse strain is prompt to develop hepatic steatosis [30]. However, further stages of NAFLD are not triggered with a high-fat diet (HFD) and fructose supplementation in drinking water is necessary [31]. Our nontargeted LC-HRMS profiling method with the Q-TOF mass spectrometer identified 719 species from 16 different lipid families. Previous lipidomic studies using LTQ-Orbitrap for mass spectrometry in liver mitochondria identified 217 species in mice fed 2 weeks [19] and 381 species in rats fed 8 weeks [18].

Mitochondrial membranes are composed of 80% of PE and PC, as well as 10-15% of CL, a component exclusive to mitochondria. Mice fed a WD for 6 to 24 weeks (45% fat and fructose in drinking water) showed an alteration of the mitochondrial lipidome with monounsaturated fatty acids [32].

Dietary-induced obesity studies in mice show changes in hepatic lipid composition, with an increase of TAG, FA, and LPC and a decrease in PE [33]. CER are typically increased and have been linked to the pathogenicity of NAFLD in association with inflammation [34]. DAG [35], CER [36, 37], and LPC [38, 39] are candidate lipotoxic species in NAFLD.

We showed that the increase of ROS was associated with lipidome alteration. The increase of ROS in NAFLD is involved in fibrosis development [15]. Interestingly, free copper was increased as well. Copper in the oxidized (Cu^2+^) state assists oxidative tissue injury through a free radical-mediated pathway [40]. In this context, Cu^2+^ may also participate in CL decrease by catalyzing its fragmentation as was demonstrated in the *Atp7b^−/−^* mouse model of Wilson's disease [41]. ROS would be aggravating the mitochondrial dysfunction itself by damaging lipids and complexes of the respiratory chain [42]. Here, a vicious circle developing between the ROS and mitochondrial lipidome changes is possible. Alteration of the oxidative phosphorylation process is a key factor in pathological ROS production [28]. The modification of mitochondrial lipidome, furthermore, among several factors involved in increasing steatosis, was highlighted as a factor for the alteration of mitochondrial structure and complex I of the respiratory chain [32].

Phospholipids such as PE and CL have a specific role in the assembly and activity of respiratory chain complexes, especially complexes III and IV, and supercomplex formation [43]. The modification of membrane lipids leads to a deficiency of respiratory complex proteins causing oxidative stress, mitochondrial damage, and insulin resistance [44]. In the context of steatosis progression, we found a significant increase in oxidative stress, associated with a decrease of CL and PE. A possibility is that the alteration of phospholipid nature, in particular CL, could be due to an overproduction of ROS. This is suggested by studies on mitochondrial dysfunction in NAFLD that showed complex I and III alterations [45] and fat accumulation [46]. We also observed that the decrease of CL is associated with an increase in ubiquinone, which could reflect an alteration of the respiratory chain. The close association between CL and ubiquinone has been previously shown to facilitate the efficiency of the electron transporter chain [13]. In a study on NAFLD in humans, an increase of CL and ubiquinone was interpreted as a compensatory mechanism [17], and our results suggest that the relationship may be more complex, depending on the stage of disease.

## 5. Conclusions

Our results show that a specific evolution of the hepatic mitochondrial lipidome typifies NAFLD natural history. Changes such as cardiolipin and CoQ antioxidant function impairment precede fibrosis, raising the possibility of a causal relationship with NAFLD progression. This specific lipid signature allows the identification of new lipid candidates in the genesis of NAFLD mitochondrial dysfunction. It could pave the way for association studies with oxidative stress or even therapeutic interventions to delay mitochondrial dysfunction. Exploration of specific mitochondrial composition alterations may offer new intervention points to treat NAFLD.

## Data Availability

The lipidomic data used to support the findings of this study are included within the supplementary information files.

## Supplementary Materials

Supplementary Materials Supplementary Table 1: details of the in-house database for lipid identification in positive and negative mode. Supplementary Table 2: list of primers for RT-qPCR. Supplementary Table 3: raw mass spectrometry data. Theoretical *m*/*z*, experimental *m*/*z*, retention time, raw lipid formula, and lipid species name. Supplementary Figure 1: quality control of mitochondria enrichment. Supplementary Figure 2: principal component analysis (PCA) of lipids in the liver.
