# Supplementary material for: Changes in Key Mitochondrial Lipids Accompany Mitochondrial Dysfunction and Oxidative Stress in NAFLD
Source: Oxid Med Cell Longev. 2021 Jun 27;2021:9986299. doi: 10.1155/2021/9986299 (PMC8257344; doi:10.1155/2021/9986299)
Supplement: Supplementary Materials — Supplementary Table 1: details of the in-house database for lipid identification in positive and negative mode. Supplementary Table 2: list of primers for RT-qPCR. Supplementary Table 3: raw mass spectrometry data. Theoretical m/z, experimental m/z, retention time, raw lipid formula, and lipid species name. Supplementary Figure 1: quality control of mitochondria enrichment. Supplementary Figure 2: principal component analysis (PCA) of lipids in the liver. [file 9986299.f1.zip › _Durand et al. suppl figures and tables v2.docx]

# Changes in key mitochondrial lipids accompany mitochondrial dysfunction and oxidative stress in NAFLD

Manon Durand^1^, Marine Coué^2^, Mikaël Croyal^2,3^, Thomas Moyon^2^, Angela Tesse^1^, Florian Atger^1^, Khadija Ouguerram^2^, David Jacobi^1^

^1^ Université de Nantes, CHU Nantes, CNRS, INSERM, l’institut du thorax, F-44000 Nantes, France

^2^ Université de Nantes, INRA, UMR 1280 Physiopathologie des Adaptations Nutritionnelles, F-44000 Nantes, France

^3^ Centre de Recherche en Nutrition Humaine Ouest, F-44000 Nantes, France

Correspondence should be addressed to

David Jacobi

Inserm UMR 1087 / CNRS UMR 6291
IRS - Université de Nantes
8 quai Moncousu
BP 70721
44007 NANTES Cedex 1

david.jacobi@univ-nantes.fr


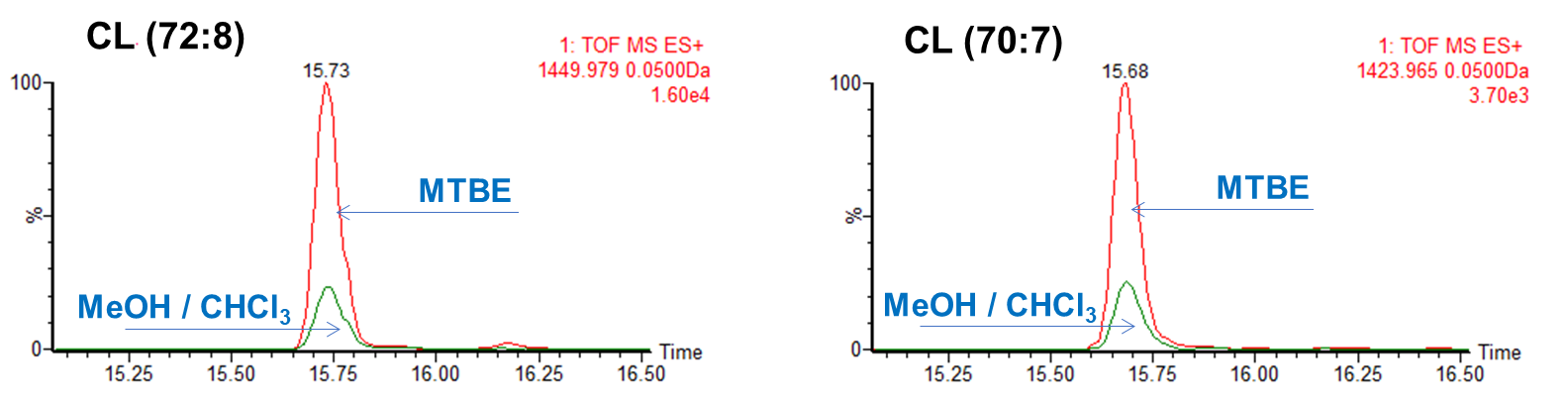


**b**

**a**


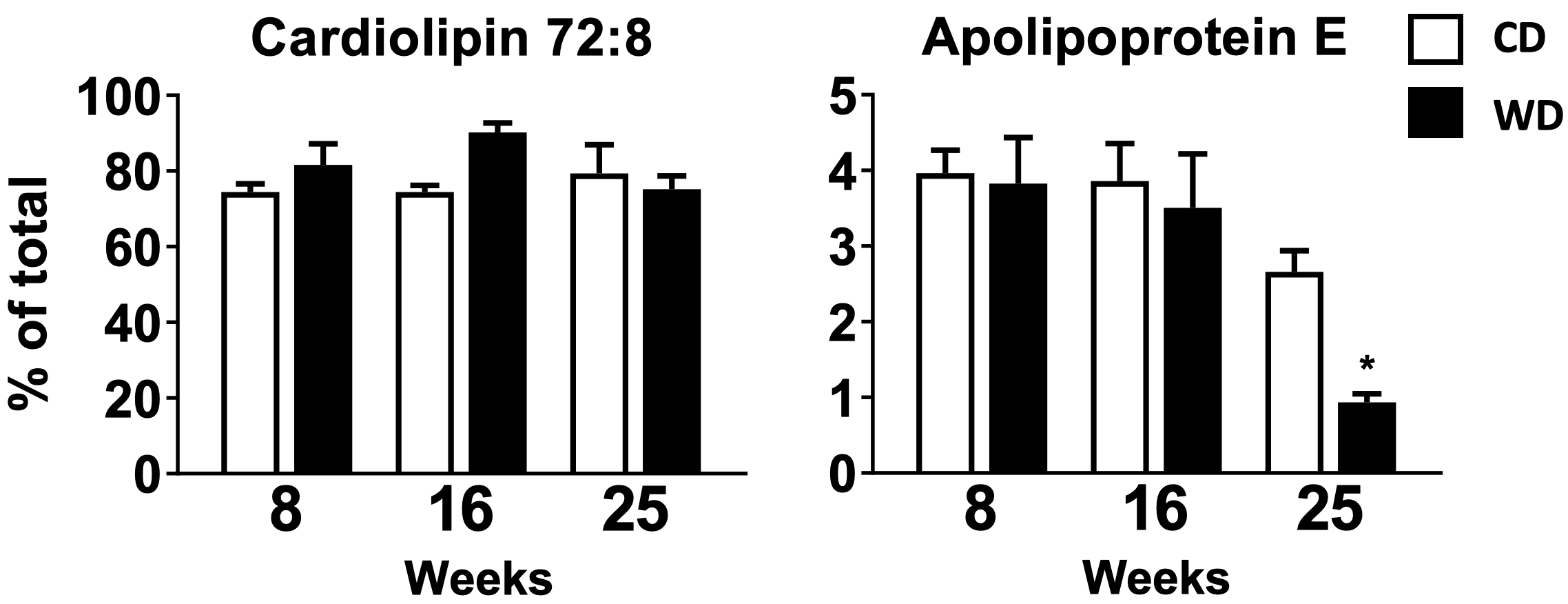


Supplementary Figure 1: Quality control of mitochondria enrichment. a. Comparison of peak intensities for two cardiolipins isolated with the methanol/chloroform method (MeOH/CHCl_3_) or the Methyl-tert-butyl-ether (MTBE) methods. b. Proportion of cardiolipin 72:8 (proxy for mitochondria retrieval) and proportion of Apolipoprotein E (proxy for mitochondria contamination with cytosolic elements) found in the mitochondria-enriched fractions.


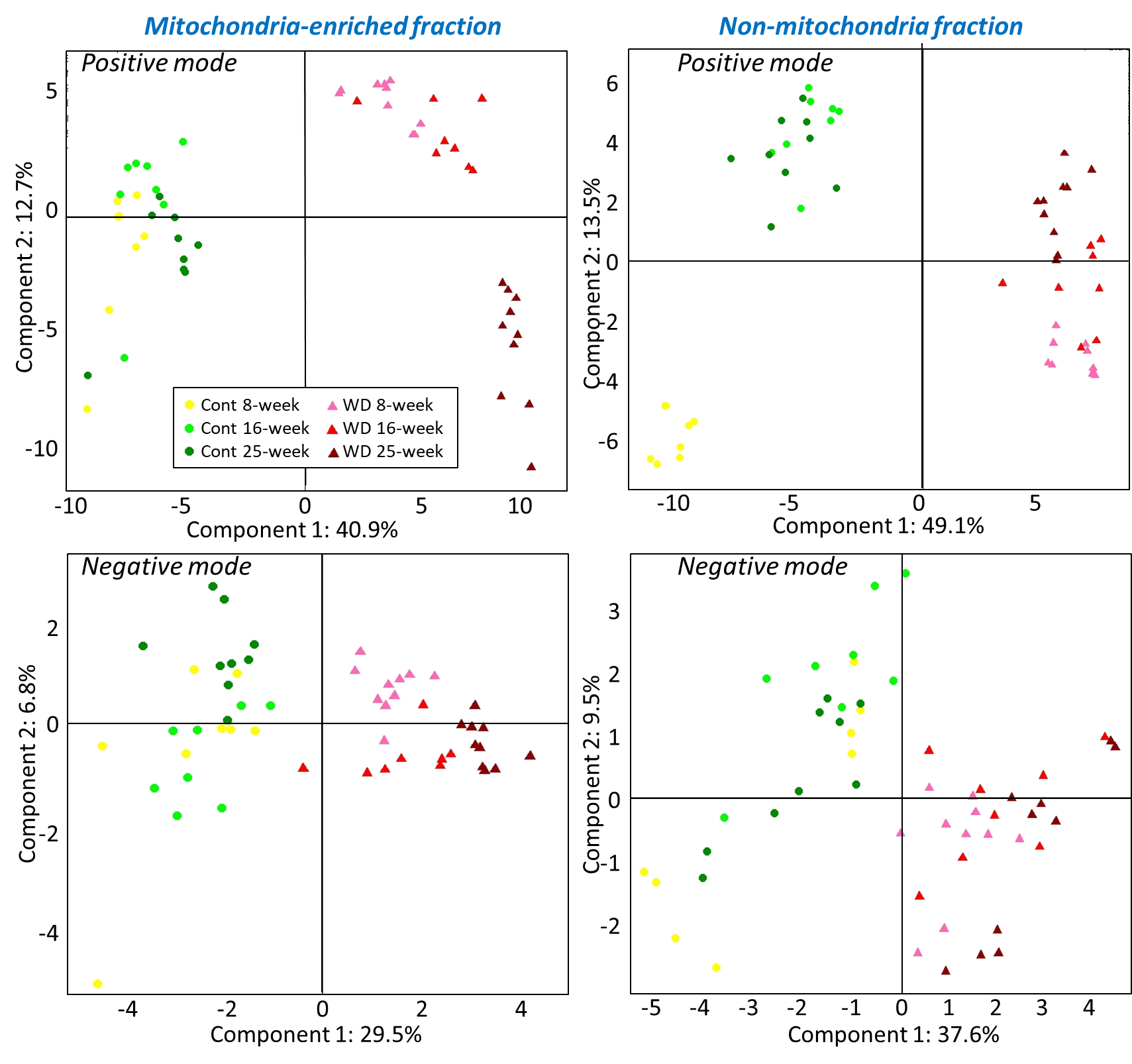


Supplementary Figure 2: Principal component analysis (PCA) of lipids in the liver. Left panels: from the mitochondrial-enriched fractions in positive mode (505 lipid species, top panel) and negative mode (53 lipid species, bottom panel). Right panels: from the non-mitochondrial fractions in positive mode (356 lipid species, top panel) and negative mode (59 lipid species, bottom panel).

| **Family** | **Species** | **N** | **Major adduct** | **Retention time (min)** |
| --- | --- | --- | --- | --- |
| Fatty acyls | FA | 12 | [M-H]^-^ | 0.86 – 5.10 |
| Glycerolipids | DG  TG | 100  156 | [M+NH_4_]^+^ / [M+Na]^+^  [M+H-H_2_0]^+^ / [M+NH_4_]^+^ / [M+H]^+^ | 15.66 – 18.89  7.87 – 18.37 |
| Glycerophospholipids | CL  LPC  LPE  LPI  PA  PC  PE  PG  PI  PS | 15  16  16  1  58  151  115  1  7  9 | [M+H]^+^ / [M+Na]^+^ / [M+H-H_2_0]^+^  [M+H]^+^ / [M+HCO_2_]^-^  [M+H]^+^ / [M-H]^-^  [M-H]^-^  [M+H-H_2_0]^+^ / [M+H]^+^ / [M+HCO_2_]^-^  [M+H]^+^ / [M+HCO_2_]^-^ / [M+H-H_2_0]^+^  [M+H]^+^ / [M-H]^-^ / [M+H-H_2_0]^+^  [M+H]^+^  [M+H]^+^  [M+H]^+^ / [M+H-H_2_0]^+^ | 9.64 – 16.35  1.11 – 2.40  1.16 – 2.59  1.04  4.98 – 18.39  6.46 – 16.82  6.88 – 16.18  10.48  6.88 – 11.43  3.63 – 15.05 |
| Sphingolipids | Cer  SM | 44  15 | [M+HCO_2_]^-^ / [M+H-H_2_0]^+^ / [M+H]^+^  [M+H]^+^ / [M+HCO_2_]^-^ | 12.03 – 16.72  8.43 – 15.64 |
| Coenzyme | CoQ | 2 | [M+H]^+^ | 15.90 – 16.82 |
| TOTAL | n/a | 718 | n/a | n/a |

Supplementary Table 1: Details of the in-house database for lipid identification in positive and negative mode. FA, fatty acid; DG, diglyceride; TG, triglyceride; CL, cardiolipin; LPC, lysophosphatidylcholine; LPE, lysophosphatidylethanolamine; LPI, lysophosphatidylinositol; PA, phosphatidic acid; PC, phosphatidylcholine; PE, phosphatidylethanolamine; PG, phosphatidylglycerol; PI, phosphatidylinositol; PS, phosphatidylserine; Cer, ceramide; SM, sphingomyelin; CoQ, Coenzyme Q.

| **Gene** | **Name** | **Forward** | **Reverse** |
| --- | --- | --- | --- |
| Srebf1 | Sterol regulatory element binding transcription factor 1 | GGAGCCATGGATTGCACATT | GGCCCGGGAAGTCACTGT |
| Ppar-α | Peroxisome proliferator-activated receptor alpha | ACAAGGCCTCAGGGTACCA | GCCGAAAGAAGCCCTTACAG |
| Tlr9 | Tool-like receptor 9 | GGGCCCATTGTGATGAACC | GCTGCCACACTTCACACCAT |
| Tnf- α | Tumor necrosis factor | ATGGCCTCCCTCTCATCAGT | TTTGCTACGACGTGGGCTAC |

Supplementary Table 2: List of primers
